# Identification of Potential Novel B-Cell Epitopes of Capsid Protein VP2 in Senecavirus A

**DOI:** 10.1128/spectrum.04472-22

**Published:** 2023-07-10

**Authors:** Yi Ru, Rongzeng Hao, Chunping Wu, Yajun Li, Bingzhou Lu, Huanan Liu, Hong Tian, Dan Li, Zhengwang Shi, Juncong Luo, Kun Ma, Guicai Zhang, Xiangtao Liu, Haixue Zheng

**Affiliations:** a State Key Laboratory for Animal Disease Control and Prevention, College of Veterinary Medicine, Lanzhou University, Lanzhou Veterinary Research Institute, Chinese Academy of Agricultural Sciences, Lanzhou, China; University of São Paulo

**Keywords:** senecavirus A, SVA, VP2 protein, immunodominant epitope, Pepscan

## Abstract

Senecavirus A (SVA) is a type of nonenveloped single-stranded, positive-sense RNA virus. The VP2 protein is a structural protein that plays an important role in inducing early and late immune responses of the host. However, its antigenic epitopes have not been fully elucidated. Therefore, defining the B epitopes of the VP2 protein is of great importance to revealing its antigenic characterization. In this study, we analyzed B-cell immunodominant epitopes (IDEs) of the VP2 protein from the SVA strain CH/FJ/2017 using the Pepscan approach and a bioinformatics-based computational prediction method. The following four novel IDEs of VP2 were identified: IDE1, ^41^TKSDPPSSSTDQPTTT^56^; IDE2, ^145^PDGKAKSLQELNEEQW^160^; IDE3, ^161^VEMSDDYRTGKNMPF^175^; and IDE4, ^267^PYFNGLRNRFTTGT^280^. Most of the IDEs were highly conserved among the different strains. To our knowledge, the VP2 protein is a major protective antigen of SVA that can induce neutralizing antibodies in animals. Here, we analyzed the immunogenicity and neutralization activity of four IDEs of VP2. Consequently, all four IDEs showed good immunogenicity that could elicit specific antibodies in guinea pigs. A neutralization test *in vitro* showed that the peptide-specific guinea pig antisera of IDE2 could neutralize SVA strain CH/FJ/2017, and IDE2 was identified as a novel potential neutralizing linear epitope. This is the first time VP2 IDEs have been identified by using the Pepscan method and a bioinformatics-based computational prediction method. These results will help elucidate the antigenic epitopes of VP2 and clarify the basis for immune responses against SVA.

**IMPORTANCE** The clinical symptoms and lesions caused by SVA are indistinguishable from those of other vesicular diseases in pigs. SVA has been associated with recent outbreaks of vesicular disease and epidemic transient neonatal losses in several swine-producing countries. Due to the continuing spread of SVA and the lack of commercial vaccines, the development of improved control strategies is urgently needed. The VP2 protein is a crucial antigen on the capsids of SVA particles. Furthermore, the latest research showed that VP2 could be a promising candidate for the development of novel vaccines and diagnostic tools. Hence, a detailed exploration of epitopes in the VP2 protein is necessary. In this study, four novel B-cell IDEs were identified using two different antisera with two different methods. IDE2 was identified as a new neutralizing linear epitope. Our findings will help in the rational design of epitope vaccines and further understanding of the antigenic structure of VP2.

## INTRODUCTION

Senecavirus A (SVA) is a type of nonenveloped single-stranded, positive-sense RNA virus belonging to the family *Picornaviridae*. In 2002, the first SVA isolate, SVV-001, was discovered in Maryland in the United States (1). SVV-001 is considered an oncolytic virus and has been utilized to treat medulloblastoma in the initial stages (2). Since 2007, the association of SVA with idiopathic vesicular disease was first identified in Manitoba, Canada (3), and SVA has become endemic in many countries, including Canada, the United States, Brazil, Thailand, Colombia, and China (4–8). From 2015 to 2020, there was an increasing number of SVA outbreaks in many different regions of China, which resulted in significant economic losses (9–13). The signature clinical sign of SVA is vesicular lesions on the nose and coronary band, leading to anorexia, lameness, and lethargy in 10% to 90% of pigs (9). Moreover, piglets within 1 week of age demonstrated a variety of signs, including weakness, salivation, cutaneous hyperemia, neurologic signs, diarrhea, and sudden death (14). The lesions of SVA infection are clinically indistinguishable from those of other infections in swine, such as foot-and-mouth disease, swine vesicular disease, and vesicular stomatitis (11). Several laboratory diagnostic methods have been developed for the detection of SVA infection, including indirect enzyme-linked immunosorbent assay (iELISA), competitive ELISA, and two specific real-time reverse transcription-PCR methods (9, 14–17). However, no existing vaccines have been licensed to limit the spread of SVA. An inactivated SVA vaccine could induce high neutralizing antibody titers in pigs after one dose of vaccination and showed good protective efficacy against SVA infection in our previous report (16).

The SVA genome is approximately 7.2 kb and includes a single open reading frame (ORF) that encodes a polypeptide (17). The single ORF follows the standard gene layout of picornaviruses, with the order 5′-L-VP4-VP2-VP3-VP1-2A-2B-2C-3A-3B-3C-3D-3′ (14). The SVA virions are mainly composed of capsids formed by four structural proteins, namely, VP1, VP2, VP3, and VP4, and their genomic RNA, which constitute the icosahedral structure responsible for infectivity (18). The capsid proteins VP1, VP2, and VP3 are located on the outer surface of the capsid of SVA. Humoral immunity mediated by neutralizing antibodies plays a critical role in *Picornavirus* infection (19). Therefore, the neutralization of SVA might be mediated through antigenic epitopes located mainly on the surface of the external viral capsid proteins (VP1, VP2, and VP3). These proteins may represent potential candidates for the development of novel vaccine formulations. Previous data indicated that VP2 plays a major role in early IgM-mediated neutralization and IgG-mediated neutralization. Moreover, the IgG titer of VP2 is approximately twice that of VP3, and antibodies to VP2 last longer than those to VP1 and VP3 (19). Therefore, the VP2 protein could be a promising candidate target for the development of novel epitope-based vaccines and suitable epitope-based diagnostic tools for SVA.

Antigenic complexes could include protective B-cell and T-cell epitopes and exclude potentially immunosuppressive and immunopathogenic determinants (20–23). Epitope-based vaccines containing selected immunogenic targets may represent an alternative and novel strategy to combat SVA (24, 25). Considering the importance of the VP2 protein in adaptive immune responses in pigs infected with SVA, a detailed exploration of epitopes in the VP2 protein is necessary (19). In this study, we defined four linear immunodominant epitopes (IDEs) of the VP2 protein in SVA strain CH/FJ/2017 using Pepscan and bioinformatic prediction methods and further analyzed their antigenicity and immunogenicity. A neutralization test indicated that IDE2 (amino acids [aa] 145 to 160) could induce a neutralizing antibody response against SVA. The results will help in the rational design of epitope vaccines by furthering the understanding of the antigenic structure of VP2 and will contribute to the development of diagnostic tools for the specific serological diagnosis of SVA infection.

## RESULTS

### Structure and quality assessment of the VP2 protein *in silico*.

**FIG 1:**
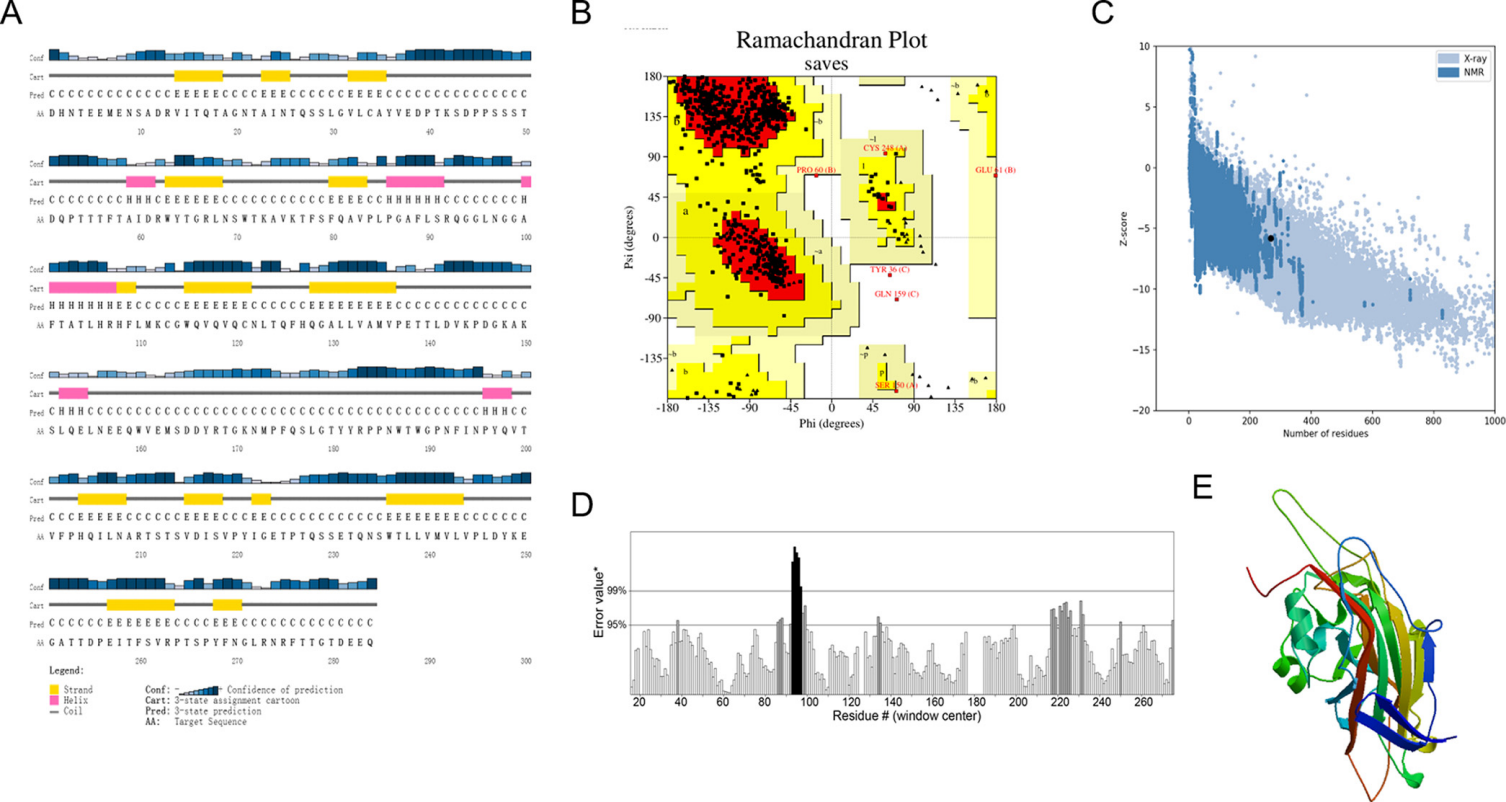
Structural and quality assessments of the VP2 protein. (A) Secondary structure features of the VP2 protein by PSIPRED software. (B) Ramachandran plot for the validation of the VP2 protein 3D structure. Yellow, the most favored regions; red, the maximum allowed regions; white, disallowed regions. (C) ProSA analysis of the VP2 protein 3D structure with Z score. A Z score of −5.8 indicated that the 3D structure of the VP2 protein had a score within the range of scores usually found for native proteins of similar size. (D) Error values for residues predicted by ERRAT, which represent the number of nonbonding interactions formed between pairs of different atomic types (side chains). The overall quality of the 3D structure shown in the ERRAT result was 87.149%. (E) Homology 3D structure of the VP2 protein.

The primary structure of the VP2 protein was analyzed by ProtParam online software (http://web.expasy.org/protparam/). The VP2 protein consists of 284 amino acid residues, with a molecular weight (MW) of 31.71 kDa and a molecular formula of C_1406_H_2145_N_377_O_443_S_9_. The theoretical isoelectric point (PI) is 5.03. The instability index of the protein is 34.57 (<40). The protein may be relatively stable. The grand average of hydrophobicity (GRAVY) value of −0.501 (<0) indicates the hydrophilicity of the protein. The secondary structure of the protein was predicted through the online secondary structure analysis software PSIPRED (http://bioinf.cs.ucl.ac.uk/psipred/). VP2 consists of 8.10% аlpha-helix regions, 24.30% beta-sheet regions, and 67.60% random coils (Fig. 1A).

Through template selection, sequence alignment, model establishment, and quality assessment, the three-dimensional (3D) homology structure of the VP2 protein was established by SWISS-MODEL (https://www.swissmodel.expasy.org/) based on template 3CJI (PDB). The stereochemistry and quality of the 3D structure were evaluated by several structural assessment methods, including Ramachandran plot, Z score, and ERRAT analyses. In the Ramachandran plot presenting the relationship between the two dihedral angles ϕ and ψ of the protein residues, 87.2% of the amino acid residues of the VP2 protein were in the most favored region, 12.1% of the residues were in additional allowed regions, 0.4% of the residues were in generously allowed regions, and 0.3% of the residues were in disallowed regions (Fig. 1B). A Z score of −5.8 indicated that the 3D structure of the VP2 protein had a score within the range of scores usually found for native proteins of similar size (Fig. 1C). The overall quality of the 3D structure shown in the ERRAT result was 87.149% (Fig. 1D). The 3D structure of the VP2 protein was visualized by Discovery Studio Visualizer Client (Fig. 1E).

### Expression and purification of the recombinant VP2 protein.

**FIG 2:**
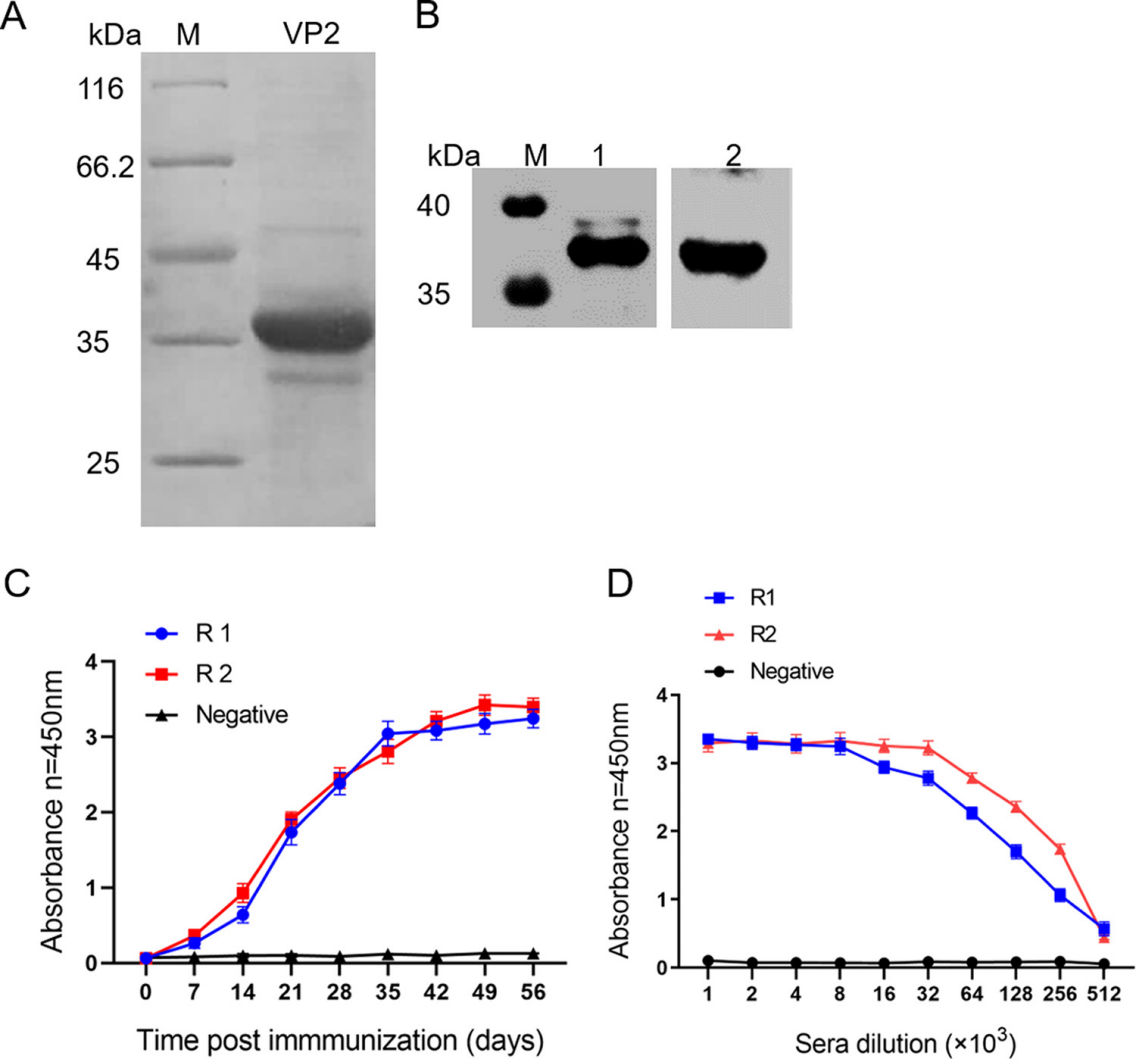
Characterization of recombinant VP2 protein and its immune serum. (A) SDS-PAGE analysis of purified recombinant VP2 protein. (B) Western blot analysis of purified recombinant VP2 protein with anti-His MAb (lane 1) and with SVA antiserum (lane 2). (C) Induced antibody levels against VP2 protein were detected by iELISA. (D) The titer of recombinant VP2 protein in immune serum was detected by iELISA. Error bars indicate the standard error of the mean (SEM) of results from three individual experiments. R1, R2, and Negative, rabbits R1 and R2 and a negative control, respectively.

Recombinant VP2 protein fused to a His tag was successfully expressed in Escherichia coli BL21(DE3). Purified recombinant VP2 protein was identified by sodium dodecyl sulfate-polyacrylamide gel electrophoresis (SDS-PAGE), and the MW of the VP2 protein was approximately 36 kDa (Fig. 2A). Western blot analysis was performed using SVA-positive pig sera and anti-His monoclonal antibody (MAb). The results showed that the VP2 protein was detected, and the size was as expected (Fig. 2B). The results demonstrated that we obtained high-purity recombinant VP2 protein that could be used to prepare polyclonal antibodies by immunizing rabbits.

### Generation and characterization of polyclonal antibodies against VP2.

Polyclonal antibodies are prepared by immunizing rabbits with purified recombinant VP2 protein. The antibody levels were determined at different time points by an iELISA. The results indicated that antibody production was induced at 14 days postimmunization (dpi) in rabbits, and the antibody level increased rapidly after 21 dpi (Fig. 2C). Rabbit sera were collected 14 days after the third immunization, and the titer of the sera was analyzed by iELISA after serial dilution. The results showed that the sera had a high binding titer (Fig. 2D).

The specificity of the rabbit sera against the VP2 protein was determined by Western blotting. A specific band corresponding to the recombinant VP2 protein (36 kDa) was found in anti-VP2 rabbit sera, and no reaction with the VP1 and VP3 proteins was observed (see Fig. S1A in the supplemental material). The indirect immunofluorescence assay (IFA) results showed that the anti-VP2 sera could specifically detect the expression of viral protein in IBRS-2 cells infected with the SVA CH-FJ-2017 strain (Fig. S1B).

### Prediction of B-cell epitopes.

The linear epitopes of the VP2 protein were predicted by using seven bioinformatic computational prediction tools (Bcepred, ABCpred, BepiPred, IEDB, SVMTriP, ElliPro, and DisoTope). The potential epitopes were sequences that were consistently identified according to the predicted results of the different bioinformatic tools. Because B-cell epitopes are usually located on the surface of proteins and in coil regions, the secondary structures and 3D structure of the VP2 protein were employed in further epitope mapping. A total of 11 potential epitope peptides were analyzed: B2-1, aa 5 to 16; B2-2, aa 35 to 57; B2-3, aa 57 to 72; B2-4, aa 70 to 79; B2-5, aa 90 to 97; B2-6, aa 145 to 160; aa B2-7, aa 160 to 175; B2-8, aa 175 to 187; B2-9, aa 220 to 235; B2-10, aa 243 to 257; and B2-11, aa 267 to 282. Then, GRAVY values were measured by the ProtParam online tool. The GRAVY values were less than 0, indicating that all the potential epitopes were located in the hydrophilic regions (Table 1).

**TABLE 1:** Predicted B-cell epitopes on the VP2 protein

Epitope	Amino acid sequence	Position (aa)	Secondary structure	Spatial position	GRAVY value
B2-1	EEMENSADRVIT	5–16	25.0% β-sheets, 75.0% coils	Exposed	−0.925
B2-2	AYVEDPTKSDPPSSSTDQPTTTF	35–57	4.3% β-sheets, 95.7% coils	Exposed	−1.174
B2-3	FTAIDRWYTGRLNSWT	57–72	25.0% β-sheets, 75.0% coils	Exposed	−0.594
B2-4	SWTKAVKTFS	70–79	20.0% β-sheets, 80.0% coils	Exposed	−0.292
B2-5	LSRQGGLN	90–97	25.0% α-helix, 75.0% coils	Exposed	−0.688
B2-6	PDGKAKSLQELNEEQW	145–160	62.5.0% α-helix, 37.5% coils	Exposed	−1.663
B2-7	WVEMSDDYRTGKNMPF	160–175	25.0% α-helix, 75.0% coils	Exposed	−1.081
B2-8	FQSLGTYYRPPNW	175–187	100.0% coils	Exposed	−1.038
B2-9	PYIGETPTQSSETQNS	220–235	100.0% coils	Exposed	−1.400
B2-10	LVPLDYKEGATTDPE	243–257	13.3% β-sheets, 86.7% coils	Exposed	−0.707
B2-11	PYFNGLRNRFTTGTDE	267–282	12.5% β-sheets, 87.5% coils	Exposed	−1.212

### Identification of VP2 B-cell epitopes using two different sera.

**FIG 3:**
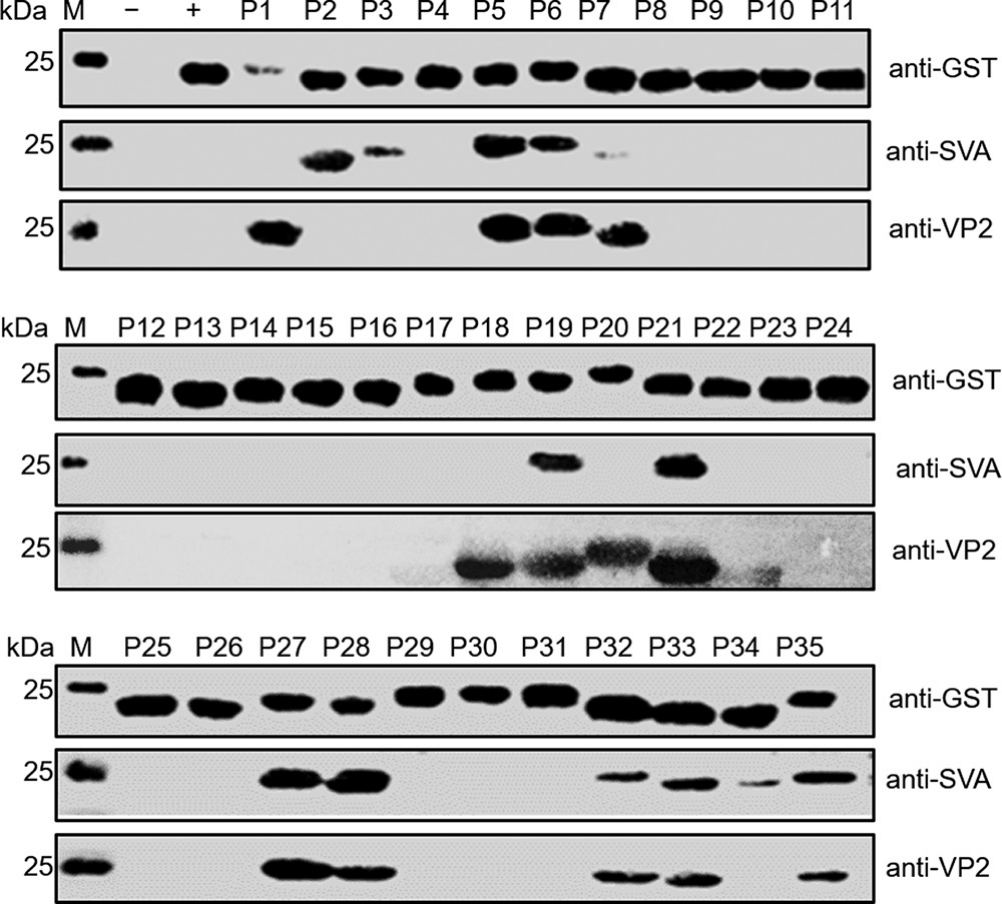
Identification of VP2 protein linear epitopes by Pepscan. The 35 overlapping epitope peptides mapping to the VP2 protein were detected by Western blotting with an anti-His MAb. Screening of epitope peptides was done using SVA antisera and VP2 antisera. Lanes P1 to P35, epitope peptides 1 to 35; lane M, protein marker; lane −, lysates of cells containing pXXGST-1 induced with IPTG; lane +, lysates of cells containing pXXGST-1 induced with IPTG.

To identify the B-cell epitopes by the Pepscan method, 35 16-mer polypeptide fragments with a truncated glutathione S-transferase (GST) gene encoding 188 residues tag were first expressed and analyzed by Western blotting (Fig. 3) (Table 2). Subsequently, the positive peptides were screened by Western blotting with VP2 antiserum and SVA antiserum as primary antibodies. Thirteen polypeptide fragments were identified by using VP2 antiserum (P1, P5, P6, P7, P18, P19, P20, P21, P27, P28, P32, P33, and P35), which indicated six restricted epitopes spanning aa 1 to 16, aa 41 to 56, aa 145 to 168, aa 217 to 224, aa 257 to 264, and aa 273 to 284, respectively (Fig. 3). Another 13 polypeptide fragments were also identified by using SVA antiserum (P2, P3, P5, P6, P7, P19, P21, P27, P28, P32, P33, P34, and P35), which indicated six restricted epitopes spanning aa 17 to 24, aa 41 to 56, aa 145 to 160, aa 161 to 176, aa 217 to 224, and aa 257 to 280, respectively. The polypeptide fragments P5, P6, P7, P19, P21, P27, P28, P32, P33, and P35 could be identified simultaneously by the two sera, which indicated that five identical sequences were identified (VP2-1, aa 41 to 56; VP2-2, aa 145 to 160; VP2-3, aa 161 to 176; VP2-4, aa 217 to 224; and VP2-5, aa 257 to 280) (Fig. 3) (Table 3). However, polypeptides P7 and P34 showed weak reactivity with the anti-SVA serum.

**TABLE 2:** Amino acid sequences of overlapping peptides for Pepscan analysis

Peptide	Amino acid sequence	Positions (aa)
P1	DHNTEEMENSADRVIT	1–16
P2	NSADRVITQTAGNTAI	9–24
P3	QTAGNTAINTQSSLGV	17–32
P4	NTQSSLGVLCAYVEDP	25–40
P5	LCAYVEDPTKSDPPSS	33–48
P6	TKSDPPSSSTDQPTTT	41–56
P7	STDQPTTTFTAIDRWY	49–64
P8	FTAIDRWYTGRLNSWT	57–72
P9	TGRLNSWTKAVKTFSF	65–80
P10	KAVKTFSFQAVPLPGA	73–88
P11	QAVPLPGAFLSRQGGL	81–96
P12	FLSRQGGLNGGAFTAT	89–104
P13	NGGAFTATLHRHFLMK	97–112
P14	LHRHFLMKCGWQVQVQ	105–120
P15	CGWQVQVQCNLTQFHQ	113–128
P16	CNLTQFHQGALLVAMV	121–136
P17	GALLVAMVPETTLDVK	129–144
P18	PETTLDVKPDGKAKSL	137–152
P19	PDGKAKSLQELNEEQW	145–160
P20	QELNEEQWVEMSDDYR	153–168
P21	VEMSDDYRTGKNMPFQ	161–176
P22	TGKNMPFQSLGTYYRP	169–184
P23	SLGTYYRPPNWTWGPN	177–192
P24	PNWTWGPNFINPYQVT	185–200
P25	FINPYQVTVFPHQILN	193–208
P26	VFPHQILNARTSTSVD	201–216
P27	ARTSTSVDISVPYIGE	209–224
P28	ISVPYIGETPTQSSET	217–232
P29	TPTQSSETQNSWTLLV	225–240
P30	QNSWTLLVMVLVPLDY	233–248
P31	MVLVPLDYKEGATTDP	241–256
P32	KEGATTDPEITFSVRP	249–264
P33	EITFSVRPTSPYFNGL	257–272
P34	TSPYFNGLRNRFTTGT	265–280
P35	RNRFTTGTDEEQ	273–284

**TABLE 3:** Immunodominant linear epitopes identified on the VP2 protein with two methods^*a*^

Peptide	Residues	Peptide sequence
Pepscan identification		
VP2-1	**41**–**56**	**TKSDPPSSSTDQPTTT**
VP2-2	**145–160**	**PDGKAKSLQELNEEQW**
VP2-3	**161–176**	**VEMSDDYRTGKNMPF**Q
VP2-4	217–224	ISVPYIGE
VP2-5	**257-280**	EITFSVRPTS**PYFNGLRNRFTTGT**
Bioinformatic identification		
B2-1	5–16	EEMENSADRVIT
B2-2	**35–57**	AYVEDP**TKSDPPSSSTDQPTTT**F
B2-4	70–79	SWTKAVKTFS
B2-6	**145–160**	**PDGKAKSLQELNEEQW**
B2-7	**160–175**	W**VEMSDDYRTGKNMPF**
B2-10	243–257	LVPLDYKEGATTDPE
B2-11	**267–282**	**PYFNGLRNRFTTGT**DE

a The residues highlighted in bold are overlapping epitopes.

**FIG 4:**
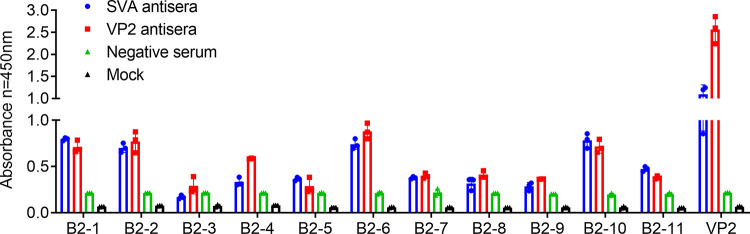
Binding analysis of the B-cell epitopes with SVA antisera and VP2 protein antiserum. The predicted B-cell epitopes were identified with the SVA antisera and VP2 protein antisera by iELISA. Unimmunized serum was used as a negative control. Each sample was detected in triplicate. Error bars represent standard deviations (SD). The S/CO value was calculated as the ratio of the mean OD value obtained with the test sample to the mean OD value of the negative control minus the mean OD value of the mock sample. An S/CO value of ≥2.1 was considered positive.

To identify the bioinformatics computationally predicted VP2 B-cell epitopes, the 11 predicted epitopeptides were chemically synthesized and assessed for MW and purity by Sangon Biotechnology (Shanghai, China) (Table 1). Eleven potential B-cell epitope peptides were further analyzed and identified by iELISA. The screening result with the SVA antiserum and the VP2 antiserum is shown in Fig. 4. Four epitopes, namely, B2-1 (aa 5 to 16), B2-2 (aa 35 to 57), B2-6 (aa 145 to 160), and B2-10 (aa 243 to 257), were judged to have a significantly high positive response to the SVA antiserum, and three epitopes, namely, B2-4 (aa 70 to 79), B2-7 (aa 175 to 187), and B2-11 (aa 267 to 282), exhibited a weakly positive reaction to the SVA antiserum. In addition, five epitopes, namely, B2-1 (aa 5 to 16), B2-2 (aa35 to 57), B2-4 (aa 70 to 79), B2-6 (aa 145 to 160), and B2-10 (aa 243 to 257), showed strongly positive reactions to the VP2 antiserum. Another four epitopes, namely, B2-7 (aa 160 to 175), B2-8 (aa 175 to 187), B2-9 (aa 220 to 235), and B2-11 (aa 267 to 282), showed a weak response to this serum. A total of seven peptides (B2-1, B2-2, B2-4, B2-6, B2-7, B2-10, and B2-11) had positive reactions to both the SVA antiserum and the VP2 antiserum (Fig. 4) (Table 3).

### Analysis of identified VP2 B-cell epitopes.

Five and seven different epitope peptides were identified by the Pepscan method and by the bioinformatic prediction method, respectively (Table 3). Based on the screening results of these two methods, VP2-1 (aa 41 to 56, **TKSDPPSSSTDQPTTT**) and B2-2 (aa 35 to 57, AYVEDP**TKSDPPSSSTDQPTTT**F) had overlapping sequences (bold). Additionally, VP2-2 (aa 145 to 160, **PDGKAKSLQELNEEQW**) had a sequence identical to that of B2-6; VP2-3 (aa 161 to 176, **VEMSDDYRTGKNMPF**Q) and B2-7 (aa 160 to 175, W**VEMSDDYRTGKNMPF**) had overlapping sequences (bold); VP2-5 (aa 257 to 280, EITFSVRPTS**PYFNGLRNRFTTGT**) and B2-11 (aa 267 to 282, **PYFNGLRNRFTTGT**DE) showed overlapping sequences (bold). Consequently, four novel epitope peptides were identified by removing the nonoverlapping amino acid residues, which were named IDE1 (^41^TKSDPPSSSTDQPTTT^56^), IDE2 (^145^PDGKAKSLQELNEEQW^160^), IDE3 (^161^VEMSDDYRTGKNMPF^175^), and IDE4 (^267^PYFNGLRNRFTTGT^280^) and were first identified in this study. Moreover, another four potential peptides were also revealed: VP2-4 (aa 217 to 224, ISVPYIGE), B2-1 (aa 5 to 16, EEMENSADRVIT), B2-4 (aa 70 to 79, SWTKAVKTFS), and B2-10 (aa 243 to 257, LVPLDYKEGATTDPE). Among these potential peptides, four epitope peptides, B2-1 (aa 5 to 16), B2-4 (aa 70 to 79), B2-10 (aa 243 to 257), and VP2-2 (aa 145 to 160), have partial sequences that overlap those previously reported (26).

### Homology modeling of the four novel identified VP2 epitopes.

**FIG 5:**
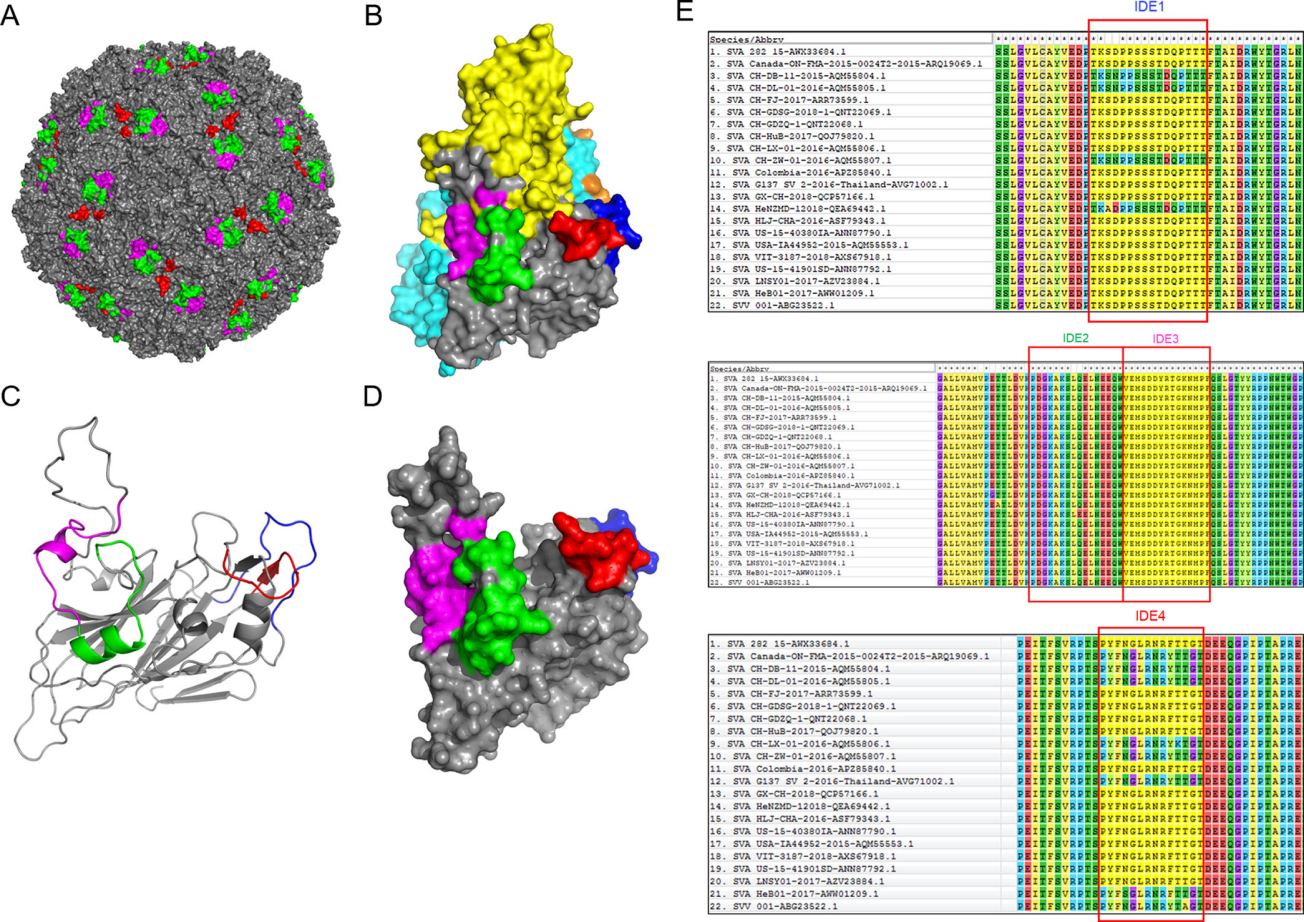
Localization of the four identified linear epitopes in the predicted 3D models of the virion and VP2 protein monomer. (A) Stereostructural analysis of the four identified epitopes in the complete SVA virion, with IDE1 (aa 41 to 56) shown in blue, IDE2 (aa 145 to 160) shown in green, IDE3 (aa 161 to 175) shown in magenta, and IDE4 (aa 267 to 280) shown in red. (B) Stereostructures of the four epitopes in the complete capsid protein (VP1 shown in yellow, VP2 shown in gray, VP3 shown in cyan, and VP4 shown in orange). (C) Secondary structural localization of the four identified epitopes presented in cartoons of the VP2 monomer skeleton. (D) Stereostructures of the four epitopes in the VP2 protein monomer. (E) Amino acid alignment was performed with different SVA isolate VP2 proteins using MEGA 7 software. The sequences of IDE1 to IDE4 are shown inside the red outlines.

The spatial locations of the identified SVA VP2 epitopes were mapped by 3D homology modeling of the SVA virion and the VP2 monomer. The results demonstrated that IDE2 (green), IDE3 (magenta), and IDE4 (red) are exposed on the surface of the virion homology model (Fig. 5A). IDE1 (blue), IDE2 (green), IDE3 (magenta), and IDE4 (red) are located on the surface of the complete viral capsid protein (VP1 shown in yellow, VP2 shown in gray, VP3 shown in cyan, and VP4 shown in orange) (Fig. 5B), and IDE2 and IDE3 form random coil and alpha-helix secondary structures (Fig. 5C). IDE4 formed random coil and beta-pleated sheet secondary structures (Fig. 5C). The epitope IDE1 (blue) is located inside the virion capsid protein, with random coil secondary structures (Fig. 5A and C). The positions of IDE1 (blue), IDE2 (green), IDE3 (magenta), and IDE4 (red) are located on the surface of the VP2 protein (VP2 shown in gray) (Fig. 5D).

### Homology analysis of the novel VP2 epitopes.

To evaluate the degree of conservation of the four novel linear epitopes identified on the SVA VP2 protein, the amino acid sequences of 22 SVA strain isolates from different countries and regions were aligned by MEGA software (Fig. 5E). We found that the different SVA strains contained few amino acid substitutions; for example only Ser at residue 43 (^43^S) in IDE1 epitope ^41^TKSDPPSSSTDQPTTT^56^ was replaced with Ala (A) in SVA strain HeNZMD-1-2018 and the Asp at residue 44 (^44^D) in this epitope was replaced with Asn (N) in SVA strains CH-ZW-01-2016, CH-DL-01-2016, and CH-DB-11-2015. Except for Gln at residue 153 (^153^Q) in the IDE2 epitope ^145^PDGKAKSLQELNEEQW^160^, which was replaced by Glu (E) in strain HLJ-CHA-2016, this epitope was completely conserved in all the SVA strains (Fig. 5E). Phe at residue 276 (^276^F) in IDE4 epitope ^267^PYFNGLRNRFTTGT^280^ was replaced with Tyr (Y) in SVA strains CH-FJ-2017, CH-ZW-01-2016, CH-LX-01-2016, CH-DL-01-2016, CH-DB-11-2015, G137-SV-2-2016, and SVV-001 (Fig. 5E). In addition, Thr at residues 277 (^277^T) and 278 (^278^T) was replaced by Lys (K) and Ala (A) in this epitope of SVA strains CH-LX-01-2016 and SVV-001, respectively (Fig. 5E). These results demonstrate that the IDE3 epitope ^161^VEMSDDYRTGKNMPF^175^ is completely conserved among SVA epidemic strains. The epitopes IDE1 and IDE2 are relatively well conserved among SVA epidemic strains.

### Immunogenicity of IDEs.

**FIG 6:**
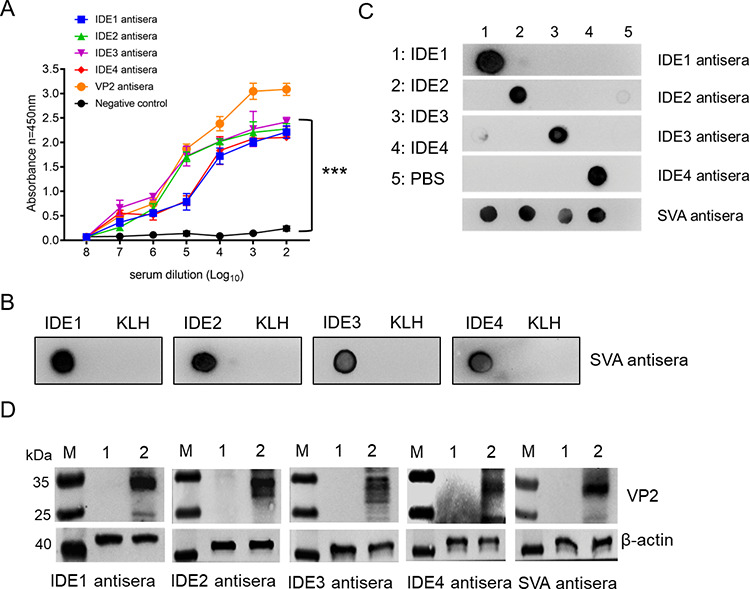
Immunogenicity of the IDE peptides. (A) Antibody titers of anti-IDE guinea pig sera and anti-VP2 rabbit serum by ELISA. Nonimmunized guinea pig serum was used as a negative control. Representative data, shown as the means ± SDs (*n* = 3), were analyzed by a two-tailed Student *t* test. ***, *P < *0.001. (B) Dot blot analysis. The IDE synthetic peptides (1 μg) were dropped on a nitrocellulose membrane and incubated with the SVA pig antisera. KLH was used as a negative control. (C) Cross-reactivity of IDE guinea pig antisera by dot blot analysis. 1 to 4, IDE1 to IDE4; 5, PBS used as a negative control. (D) IDE guinea pig antisera recognizing SVA. IBRS-2 cells were infected with SVA at a multiplicity of infection of 0.5 at 24 h and analyzed by Western blotting with IDE guinea pig antisera. Lanes: M, protein marker; 1, uninoculated cells used as a negative control; 2, cells infected with SVA.

To analyze the immunogenicity of the IDEs, we immunized guinea pigs with synthetic peptides. Antisera were prepared for serological analysis. The four IDEs induced high antibody titers, as shown by iELISAs (Fig. 6A), and the peptides could be recognized with the SVA antisera by dot blotting (Fig. 6B). Each antiserum did not cross-react with other IDEs (Fig. 6C), indicating the specific recognition of these antisera. Furthermore, these antisera could recognize VP2 in SVA-infected cells (Fig. 6D). These results indicated that these four IDE peptides could specifically elicit humoral responses in animals.

### The neutralizing activities of IDEs.

**FIG 7:**
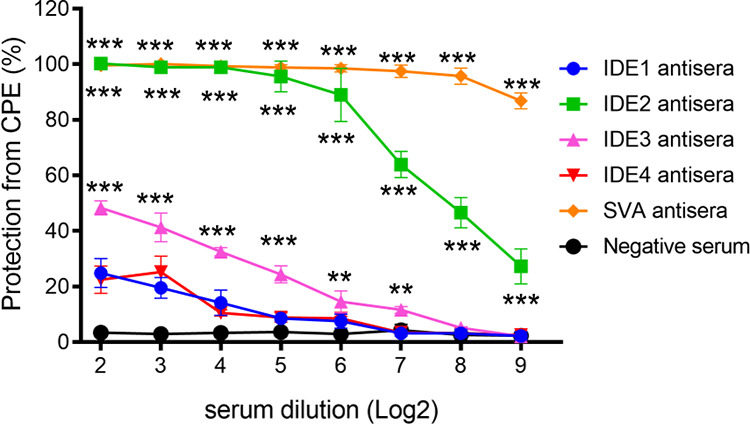
Virus-neutralizing activities of the IDE-specific antisera against SVA. The IDE guinea pig antisera were serially diluted in 96-well plates and incubated with SVA at 200 TCID_50_. Twenty-four hours after infection, cell viability was detected by the CCK-8 assay. The protection from CPE (%) was determined as follows: 100 × (OD 450 of each serum – OD 450 of PC)/(OD 450 of cell control – OD 450 of PC), with PC representing the virus control. Asterisks indicate the results of a two-tailed Student *t* test between IDE1 antisera and another IDE serum or SVA antisera, and a *P* value of <0.05 was considered statistically significant (*). Meanwhile, *P* values of <0.01 and <0.001 are marked with two and three asterisks, respectively.

VP2 antibodies possessing neutralizing activities can effectively block SVA infection (25). To investigate whether the IDE-specific antisera had virus-neutralizing activity, a virus neutralization assay was performed. The anti-SVA pig sera as a positive control could significantly inhibit SVA infection at high dilutions. The percentage of protection from CPE was still more than 80%, even though the dilution of the sera was 2^9^ (1:512). The dilution of IDE2 antisera at 2^2^ to 2^5^ (1:4 to 1:32) showed obvious inhibition of SVA infection similar to that of the anti-SVA pig sera (Fig. 7). The antisera of IDE2 showed partial inhibition of SVA infection at dilutions of 2^6^ to 2^9^ (1:64 to 1:512). The neutralizing activities of the IDE2 antisera gradually decreased, with the percentages of protection from CPE at 95%, 88%, 63%, and 46% at dilutions of 2^5^ to 2^8^ (1:32 to 1:256). In addition, the neutralizing activities of the IDE3 antisera gradually decreased, with the percentages of protection from CPE at 48%, 41%, 32%, and 24% at dilutions of 2^2^ to 2^5^ (1:4 to 1:32) (Fig. 7). The antisera of IDE1 and IDE2 could not block virus infection (Fig. 7). These data indicated that the antisera of IDE2 showed partial virus-neutralizing activities against virulent SVA strain CH/FJ/2017. Taken together, these results suggest that IDE2 may contain a virus-neutralizing epitope for SVA. These results were also confirmed by a previous report (25, 26).

## DISCUSSION

In recent years, the lack of an effective vaccine has led to an epidemic of SVA in different countries and regions, resulting in productivity impairment of finishing pigs and in the death of piglets (27). The VP2 protein plays a major role in the generation of SVA-neutralizing antibodies and long-term immune protection in pigs (25, 26). The novel epitopes identified in this study will help to elucidate the antigenic epitopes and the basis for immune responses of the VP2 protein. Fan et al. (26) identified five B-cell epitopes on the VP2 protein by using 29 overlapping synthetic peptides spanning VP2. The quality, purity, and form of the immunogen are essential for antibody development. In this study, we prepared a highly purified form of soluble recombinant VP2 protein. The pig sera with high neutralizing titers and the anti-VP2 rabbit serum were combined to map potential epitopes in the VP2 protein to improve the accuracy of epitope mapping.

The sensitivity and accuracy of the screening method are also indispensable for mapping epitopes. At present, various methods, such as homologue-scanning mutagenesis (28), the use of synthetic peptides (29), phage display of random fragment libraries (30), and computational methods (31), have usually been applied for the identification of linear B-cell epitopes. However, due to various limitations, the performance of different methods, including improved and derived methods, is far from ideal (32). The accuracy and comprehensiveness of epitope identification can be improved by exploiting the complementarity of diverse methods (31). The use of biosynthetic peptides expressing the gene fragment with *in silico* predictions is a relatively simple, effective, and reliable method for B-cell linear epitope identification and is suitable for general laboratories (33–35). Moreover, the types of antibodies in the antisera, including neutralizing and common antibodies, are more comprehensive, which is the basis for finding B-cell epitopes of all desired functions (36–39). Thirteen 16-mer peptides were identified with VP2 antisera by the Pepscan method, among which only P1 and P20 could specifically react with antisera. Another 13 positive 16-mer peptides were identified by using SVA antisera, among which only P2, P3, and P34 could specifically respond to the pig hyperimmune serum. These results may be explained by the differences among animal species having different abilities to respond to the antigen, and the antibodies produced are also different, so the viral particles presented and the epitopes identified with the animals may differ (40).

With the enormous expansion of knowledge about the 3D structure and the epitopes of many proteins, a large number of computational algorithms have been developed for mapping epitopes. Bioinformatic tools are quick and extremely efficient methods for structure identification and mapping of epitopes. However, the prediction accuracy of bioinformatic tools is limited (41); therefore, combining multiple bioinformatic tools will improve the prediction accuracy (42). A wide range of epitopes were found on the surface and in random coil regions of the proteins. Therefore, further structural screenings are absolutely essential. In this study, B-cell epitopes were predicted by 3D structure analysis combined with consensus methods using various online tools and data sets. Subsequently, the potential epitopes were further verified by iELISA. These results suggested that various epitopes, including B2-1, B2-2, B2-6, and B2-10, show extraordinary affinity with the SVA antisera and the VP2 antisera simultaneously. These identified epitopes had low GRAVY values and were almost all on the surface of the VP2 protein. Moreover, these epitopes were located in relatively hydrophilic regions and had elevated IgG binding ability. Therefore, these epitopes provided the maximum possibilities for antigen-antibody binding and were likely essential B-cell epitopes. Four IDEs presented overlapping amino acid sequences that were also identified by Fan et al. (26), namely, ^12^DRVITQT^18^ in B2-1, ^71^WTKAVK^76^ in B2-4, ^248^YKEGAT^253^ in B2-10, and ^150^KSLQELN^156^ in VP2-2.

In summary, the accuracy and comprehensiveness of epitope identification can be improved by combining multiple different methods and immunized serum. Moreover, highly specific and neutralizing antibodies can dramatically improve the reliability and practicality of the screened epitopes. In particular, the reliability of the revealed IgG epitope depends upon the preparation of antisera, and the efficiency of epitope identification is significantly improved by the application of Pepscan and bioinformatic methods. In this study, 12 linear B-cell epitopes screened against anti-VP2 rabbit sera and anti-SVA hyperimmune sera were revealed (Table 3). Importantly, three novel linear B-cell epitopes, namely, ^41^TKSDPPSSSTDQPTTT^56^, ^217^ISVPYIGE^224^, and ^267^PYFNGLRNRFTTGT^280^, were first identified on the VP2 protein. Moreover, conservation analysis of the four IDEs through sequence alignment showed that IDE3 was completely conserved in 22 epidemic SVA strains. In addition, IDE1 and IDE2 were also highly conserved among most SVA strains. However, compared to the above two epitopes, IDE4 was partly conserved. Furthermore, the IDE2 antisera at dilutions of 2^2^ to 2^5^ (1:4 to 1:32), similar to the SVA antisera, showed high inhibition of SVA in the virus neutralization assay. However, the IDE3 antisera showed slight inhibition of viruses. Although the amino acid sequences of IDE2 and IDE3 were highly conserved among the SVA strains, these two IDEs were exposed on the surface of the SVA virion. This result might be due to the difference in immunodominant sequences of envelope proteins. The differences in the epitope dominant sequences of viral proteins may cause the different immunogenicity and reactivity of the IDE.

In this study, we identified four IDEs of VP2 in SVA using the Pepscan approach combined with bioinformatic methods. The guinea pigs immunized with four IDE peptides produced IDE-specific antibodies that did not cross-react with each other. IDE2 and SVA antisera, neutralizing antibodies, present similar levels of virus-neutralizing activity in an *in vitro* assay. In summary, these results will help to understand the antigenic characteristics of VP2 and promote the development of epitope vaccines and diagnostic tools for SVA.

## MATERIALS AND METHODS

### Ethics statements.

This study was carried out in strict accordance with the recommendations in the *Guide for the Care and Use of Laboratory Animals* of the Ministry of Science and Technology of the People’s Republic of China. The animal experimental protocol was approved by the Animal Care and Ethics Committee of Lanzhou Veterinary Research Institute, Chinese Academy of Agricultural Sciences (CAAS). The procedures were carried out according to the *Guiding Principles for Biomedical Research Involving Animals*.

### Reagents and materials.

SVA strain CH/FJ/2017 (GenBank accession no. KY747510) was isolated by our laboratory. IBRS-2 cells were cultured in Dulbecco’s modified Eagle’s medium (DMEM) (Invitrogen, Carlsbad, CA, USA) containing 10% heat-inactivated fetal bovine serum (FBS) (Gibco, Grand Island, NY, USA) at 37°C in a humidified incubator under 5% CO_2_. New Zealand White rabbits and guinea pigs were provided by the Experimental Animal Centre of Lanzhou Veterinary Research Institute, CAAS. Anti-SVA hyperimmune sera, Escherichia coli TOP 10, BL21(DE3) cells, and the prokaryotic expression vectors pET-28a (+) and pXXGST-1 were stored in our laboratory. Fluorescein isothiocyanate (FITC)-conjugated goat anti-pig IgG antibody (H+L), Alexa Fluor 488-conjugated goat anti-rabbit IgG antibody (H+L), mouse anti-glutathione *S*-transferase (anti-GST) monoclonal antibody (3G10/1B3), and horseradish peroxidase (HRP)-labeled goat anti-mouse IgG, rabbit IgG, and pig IgG antibodies (Abcam, USA).

### Expression and purification of the recombinant VP2 protein.

The gene sequence of VP2 with a 6×His tag added at the 3′ end was optimized according to the codon usage bias of the E. coli expression system and synthesized by BGI Genomics Corporation (Beijing, China), which was used to insert VP2 into the pET-28a (+) expression vector. After identification of the recombinant expression plasmid pET-28a-VP2 by sequencing, it was transformed into E. coli BL21(DE3) cells, and expression was induced by 0.5 mM isopropyl β-d-1-thiogalactopyranoside (IPTG) at 16°C for 12 h. The cells were harvested by centrifugation at 4,200 rpm for 10 min and lysed on ice by an ultrasonic crushing apparatus. The precipitate and supernatant were collected by centrifugation at 18,000 rpm for 1 h. Then, the expression of recombinant VP2 protein was analyzed by SDS-PAGE.

The recombinant VP2 protein was purified using a HisTrap nickel affinity chromatography column (GE, USA) according to the manufacturer’s instructions. The purification of the VP2 fusion protein was analyzed by SDS-PAGE and Western blotting.

### Preparation of the polyclonal antibody against the VP2 protein and anti-IDE peptide guinea pig sera.

Two New Zealand White rabbits (R1 and R2) were immunized subcutaneously with 200 μg of the purified VP2 protein emulsified in complete Freund’s adjuvant at multiple sites on the rabbits’ backs. Booster immunizations were administered three times subcutaneously with 100 μg of the purified VP2 protein emulsified in incomplete Freund’s adjuvant at 2-week intervals. Rabbits immunized with phosphate-buffered saline (PBS) served as negative controls. Blood collection was started on the 7th day after the first immunization and then once a week thereafter. The serum titer level was detected through iELISA by using recombinant VP2 protein as the antigen.

Because epitope peptides are usually too short to provoke a sufficient immune response, IDE peptides were synthesized and conjugated with keyhole limpet hemocyanin (KLH) by Sangon Biotechnology (Shanghai, China). Each peptide was dissolved to 1 mg/mL with PBS. Five guinea pigs (250 g) with each peptide emulsified by complete Freund’s adjuvant (Sigma) were subcutaneously immunized with 200 μg per guinea pig. Guinea pigs immunized with PBS served as negative controls. Each animal was boosted at 3 weeks after the first immunization with the peptide emulsified by incomplete Freund’s adjuvant (Sigma). Finally, blood samples were collected at day 42. The antisera of four IDEs were prepared.

### iELISA.

iELISA based on the recombinant VP2 protein was used to identify the antibody titers. Polysorp 96-well ELISA plates were coated with 50 ng of recombinant VP2 protein in 100 μL of 0.05 M carbonated bicarbonate (pH 9.6) by overnight incubation at 4°C. After the samples were washed five times with PBST (PBS with Tween 20), the plates were blocked with 5% skim milk at 37°C for 2 h and washed with PBST. A total of 100 μL of antiserum (pig serum and rabbit serum), serially diluted 2-fold in PBS, was added to each well. The plates were incubated for 30 min at 37°C and washed with PBST. HRP-conjugated goat anti-rabbit IgG or anti-pig IgG (diluted 1:10,000) was added to each well and incubated at 37°C for 30 min. After another wash, 100 μL of 3,3′,5,5′-tetramethylbenzidine as a chromogenic substrate solution was added to each well. The reaction was stopped with 50 μL of 0.5 M H_2_SO_4_ after incubation at room temperature for 30 min. The optical density at 450 nm (OD_450_) of each well was measured using an ELISA reader.

iELISA based on synthetic peptides was utilized to screen the predicted epitopes. The iELISA method was the same as described above. The cutoff (CO) value was calculated as the mean OD of the negative control minus the mean OD of the blank well. The results are expressed as the S/CO value, which was calculated as the ratio of the OD obtained with the test sample to the cutoff value determined concurrently. An S/CO of ≥5 was scored as a strongly positive reaction, values of 2.1 to 4.9 were scored as weakly positive, and an S/CO of <2.1 was scored as a negative reaction.

### Western blotting and dot blotting.

The proteins were separated on an SDS-PAGE gel and then transferred to a nitrocellulose membrane (ISEQ00010, Merck Millipore), which was blocked in 5% skim milk at room temperature for 1 h. After the membrane was washed with PBST three times, it was incubated with individual protein-specific primary antibodies at 4°C overnight on a shaker. After washing, the membrane was incubated with HRP-conjugated IgG antibody for 1 h at room temperature. Finally, after three more washes, the membranes were treated with a chemiluminescence reagent solution kit (ECL; Thermo Scientific, USA), and the antibody-antigen complexes were exposed and detected with an imaging system (GelDocXR, Bio-Rad, USA).

The synthesized peptide (1 μg) was dropped onto a nitrocellulose membrane and incubated at 37°C for 10 min. The membrane was blocked with 5% skim milk at 37°C for 1 h. After the membrane was washed three times with TBS, it was incubated with 1:1,000 anti-IDE guinea pig serum diluted with Tris-buffered saline (TBS) at 37°C for 1 h. After the membrane was washed with TBST (TBS with Tween 20), it was incubated with the corresponding HRP-conjugated goat anti-guinea pig secondary antibody. An ECL reagent solution kit (Thermo Scientific, USA) was used for detection.

### IFA.

In total, 5 × 10^4^ IBRS-2 cells were inoculated in 35-mm laser confocal microscopy dishes and infected with 100 50% tissue culture infective doses (TCID_50_) of SVA strain CH-FJ-2017. Uninfected cells were used as a negative control. At 24 h postinfection (hpi), the infected cells were fixed with 4% paraformaldehyde for 30 min at room temperature. After the cells were washed three times with PBS, they were blocked with 4% bovine serum albumin containing 0.4% Triton X-100 in PBS at 37°C for 2 h. After the cells were washed with PBS three times, they were incubated with a primary antibody at 4°C for 12 h. After the cells were washed three times with PBS, they were incubated with secondary antibody-conjugated FITC in a moist container in the dark at 37°C for 1 h. After the cells were washed three times with PBS, they were stained with DAPI (4′,6-diamidino-2-phenylindole) for 5 min at room temperature. Finally, fluorescent images were visualized and captured on a confocal laser scanning microscope (CLSM) (Leica, Germany).

### Pepscan analysis.

To investigate the IDEs of the VP2 protein, 35 overlapping peptides spanning the entire VP2 gene sequence were expressed with a GST tag to generate fusion proteins (Table 2). All 16-mer truncated polypeptides with an overlap of 8 aa residues covering the full-length VP2 protein were designed and expressed as fusion proteins with a GST188 tag as described by Xu et al. (35). The DNA fragments encoding each 16-mer peptide were synthesized and inserted downstream of the GST188 genes in the pXXGST-1 plasmids, which all had a BamHI cohesive site at the 5′ end and a TAA-EcoRI cohesive site at the 3′ end. After verification by sequencing, the recombinant plasmids pXXGST-P1 to pXXGST-P35 were transferred into E. coli BL21(DE3) and cultured in a shaker at 37°C and 220 rpm for 4 h, and pXXGST-1 was transferred into E. coli BL21(DE3) as a control. When the OD_600_ was between 0.6 and 0.8, protein expression was induced by 0.5 mM IPTG for 12 h at 16°C. The cells were collected by centrifugation at 4,200 rpm for 10 min. The cell pellets were resuspended in PBS, lysed on ice with an ultrasonic crushing apparatus, and centrifuged at 18,000 rpm for 1 h. The precipitate and supernatant were collected, and then the fusion protein was purified using a GSTrap high-performance affinity chromatography column (GE, USA) according to the manufacturer’s instructions. The expression of 16-mer polypeptide fragments was analyzed by SDS-PAGE and Western blotting. Then, peptide mapping analysis was performed with anti-SVA pig hyperimmune serum and anti-VP2 rabbit-immunized serum in incubation buffer for 12 h at 4°C. After the membrane was washed, it was incubated with the corresponding HRP-conjugated secondary antibody. An ECL reagent solution kit (Thermo Scientific, USA) was used for detection.

### Prediction and synthesis of epitope peptides.

First, the linear epitopes of the VP2 protein were predicted using five bioinformatic software programs: Bcepred (https://webs.iiitd.edu.in/raghava/bcepred/; 12 May 2020), ABCpred (https://webs.iiitd.edu.in/raghava/abcpred/; 12 May 2020), Bepipred (https://services.healthtech.dtu.dk/services/BepiPred-3.0/; 12 May 2020), IEDB (https://www.iedb.org/, 12 May 2020), and SVMTrip (http://sysbio.unl.edu/SVMTriP/; 12 May 2020) (43). Then, the 3D structure of the VP2 protein was used to predict the conformations of B-cell epitopes of VP2 by bioinformatic software, including ElliPro (http://tools.iedb.org/ellipro/; 18 May 2020) and DisoTope (https://www.britannica.com/science/isotope; 18 May 2020.) (41). Finally, consistent epitope sequences were identified by these seven bioinformatic tools combined with secondary structure analysis and 3D structure analysis. The 11 predicted epitope peptides were synthesized and assessed for MW and purity by Sangon Biotechnology (Shanghai, China).

### Homology modeling of VP2 protein.

The amino acid sequence of the VP2 protein (GenBank accession no. KY747510) was obtained from the NCBI protein database (www.ncbi.nlm.nih.gov/). Then, the primary structure of the VP2 protein was analyzed with the ProtParam online tool (https://web.expasy.org/protparam) (44), which allows the computation of various physical and chemical parameters for a given protein stored in Swiss-Prot or TrEMBL or for a user-entered protein sequence. The computed parameters include the molecular weight, theoretical pI, amino acid composition, atomic composition, extinction coefficient, estimated half-life, instability index, aliphatic index, and grand average of hydropathicity (GRAVY). The secondary structure was analyzed with the PRIPRED online tool (http://bioinf.cs.ucl.ac.uk/psipred) (45), which provides a range of protein structure prediction methods for secondary structure prediction of proteins, including regions of disorder and transmembrane helix packing, contact analysis, fold recognition, structure modeling, and prediction of domains and function. The appropriate template of the VP2 protein was selected and employed for homology modeling of the VP2 structural protein using SWISS-MODEL (https://swissmodel.expasy.org/interactive) online software (46). Then, Discovery Studio Visualizer Client 2017R2 version software was used to visualize the 3D structure of the VP2 protein (33).

Structural evaluation and stereoscopic chemistry analysis of the VP2 protein’s 3D structure were performed using a variety of evaluation and validation tools. The backbone conformation was evaluated by analyzing the Psi/Phi Ramachandran plot (https://saves.mbi.ucla.edu/), and the result indicated the relationship between the two dihedral angles ϕ and ψ of the protein residues. The ProSA-web server (http://prosa.services.came.sbg.ac.at/prosa.php), which is an interactive web service for the recognition of errors in three-dimensional structures of proteins, was utilized to assess the Z score of the predicted VP2 3D structure (47). Further evaluation of the 3D structure was performed by ERRAT (http://services.mbi.ucla.edu/ERRAT/) (48), which is a program for verifying protein structures determined by crystallography. Error values are plotted as a function of the position of a sliding 9-residue window. The error function is based on the statistics of nonbonded atom-atom interactions in the reported structure (compared to a database of reliable high-resolution structures).

### Virus neutralization activity assays.

Briefly, different diluted antisera (anti-IDE and anti-SVA pig sera) and normal guinea pig serum as controls were used in IBRS-2 cells to perform the neutralization test. Fifty microliters of the antiserum treated at 56°C for 30 min was serially diluted 2-fold from 1:4 to 1:512. Then, the diluted antisera were mixed with SVA strain CH-FJ-2017 (200 TCID_50_) and incubated for 1 h at 37°C in a 96-well plate. Subsequently, 100 μL of the mixtures was transferred to IBRS-2 cells in a 96-well plate and incubated for 1 h at 37°C. The cell supernatant was discarded, and after three washes, DMEM containing 2% FBS was added. After incubation for 48 h, CCK-8 solution (APExBIO, USA) was added to the cells, and cell viability was detected according to the instructions of the CCK-8 assay kit. The percent protection from CPE was measured as described by Li et al. (49) with minor modifications. The protection from CPE (%) was calculated as follows: 100 × (OD 450 of each serum – OD 450 of PC)/(OD 450 of cell control – OD 450 of PC), with Positive control (PC) representing a virus infection control. Each serum sample was assayed in triplicate, and the data are presented as the average value of triplicate results.

### Homology modeling of the identified VP2 epitopes.

Homology modeling of the virion with a solid surface and the VP2 monomer was accomplished with SVV-001 (PDB ID 3CJI). The spatial and secondary structures of the four novel identified epitopes in the SVA VP2 protein were analyzed by mapping them onto the virion and onto the VP2 monomer by using the SWISS-MODEL online server and PyMol software.

### Homology analysis.

To investigate the degree of conservation of the four identified novel linear B-cell epitopes in different SVA strains, 22 SVA VP2 amino acid sequences collected from the GenBank database were aligned with the amino acid sequences of the identified epitopes in VP2 using MEGA software.

### Statistical analysis.

The experiments described above were performed in triplicate. The various treatments were compared by an unpaired, two-tailed Student *t* test while assuming unequal variance. A *P* of <0.05 was considered statistically significant (*). Meanwhile, *P* values of <0.01 and <0.001 are marked with two and three asterisks, respectively.

### Data availability.

Data and materials described in this study are available from the corresponding authors upon reasonable request.
