# Supplementary figures and images for: Identification of Potential Novel B-Cell Epitopes of Capsid Protein VP2 in Senecavirus A
Source: Microbiol Spectr. 2023 Jul 10;11(4):e04472-22. doi: 10.1128/spectrum.04472-22 (PMC10433816; doi:10.1128/spectrum.04472-22)

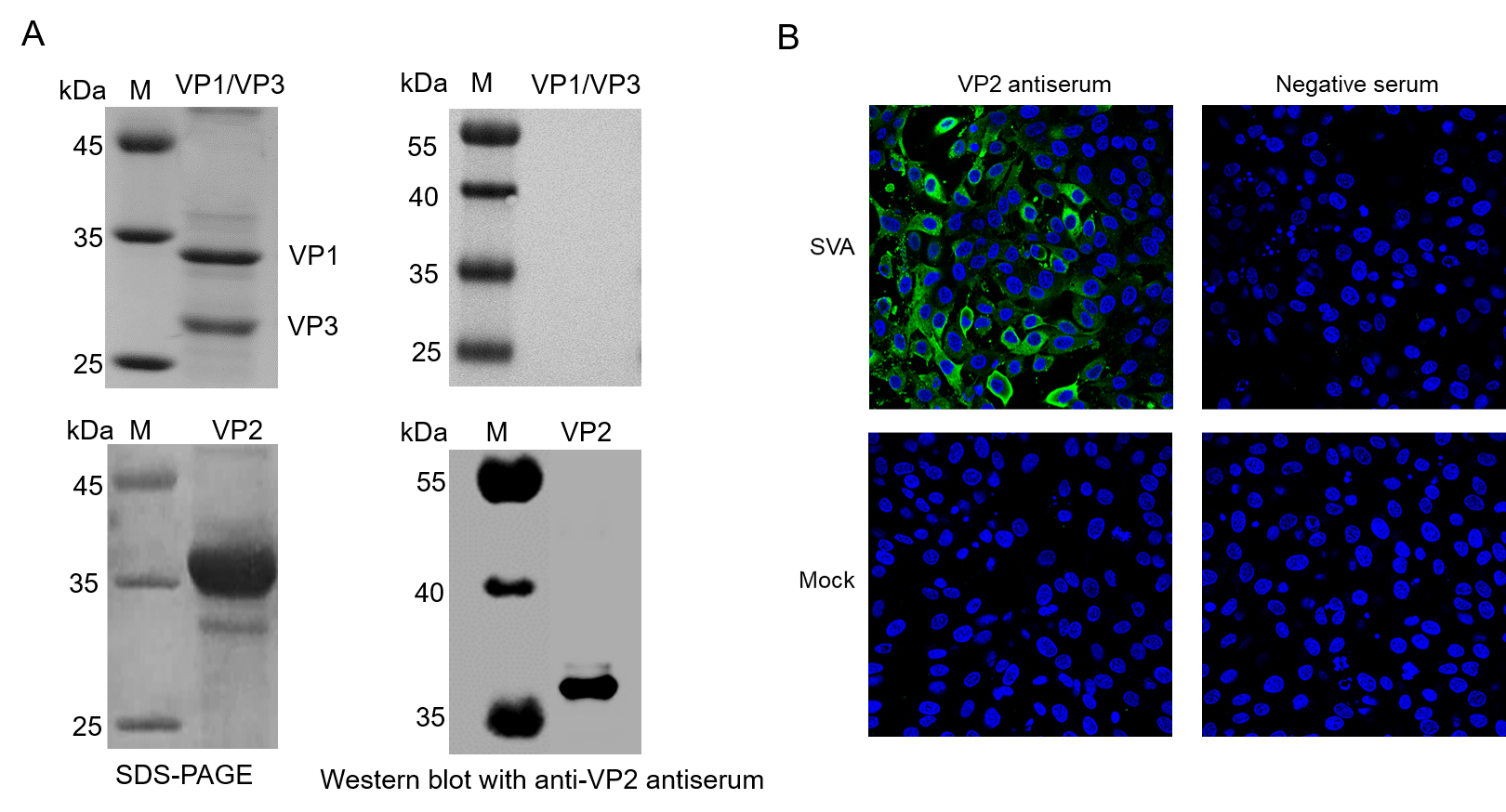

Supplement: Supplemental file 1 — Figure S1. Download spectrum.04472-22-s0001.tif, TIF file, 1.0 MB [file spectrum.04472-22-s0001.tif]
