# Supplementary material for: Identification of Potential Novel B-Cell Epitopes of Capsid Protein VP2 in Senecavirus A
Source: Microbiol Spectr. 2023 Jul 10;11(4):e04472-22. doi: 10.1128/spectrum.04472-22 (PMC10433816; doi:10.1128/spectrum.04472-22)
Supplement: Supplemental file 2 — Fig. S1 legend. Download spectrum.04472-22-s0002.docx, DOCX file, 0.03 MB [file spectrum.04472-22-s0002.docx]

**FIG S1.** Characterization and identification of VP2 antiserum. (A) Analysis of the reactivity of the VP2 antiserum by Western blot. The SVA VP1, VP2 and VP3 proteins were separated by SDS–PAGE (left). Specificity analysis of the VP2 antiserum by Western blot (right). (B) Indirect immunofluorescence assay (IFA) was used to analyse the specificity of VP2 antisera. The green colour represents VP2 protein antisera, and the blue colour represents nuclei. The mock-infected cells were used as a negative control. Scale bars, 100 μm.
